# Quantum Generative Modeling of Single-Cell transcriptomes: Capturing Gene-Gene and Cell-Cell Interactions

**Published:** 2025-12-19

**Authors:** Selim Romero, Vignesh S Kumar, Robert S. Chapkin, James J. Cai

**Affiliations:** 1 Department of Veterinary Integrative Biosciences, Texas A&M University .; 2 Department of Nutrition, Texas A&M University .; 3 CPRIT Single Cell Data Science Core, Texas A&M University .; 4 Department of Electrical and Computer Engineering, Texas A&M University .

**Keywords:** Quantum Computing, Quantum Sampler, Biophysics, Bioinformatics, Single-cell

## Abstract

Single-cell RNA sequencing (scRNA-seq) data simulation is limited by classical methods that rely on linear correlations, failing to capture the intrinsic, nonlinear dependencies. No existing simulator jointly models gene-gene and cell-cell interactions. We introduce qSimCells, a novel quantum computing-based simulator that employs entanglement to model intra- and inter-cellular interactions, generating realistic single-cell transcriptomes with cellular heterogeneity. The core innovation is a quantum kernel that uses a parameterized quantum circuit with CNOT gates to encode complex, nonlinear gene regulatory network (GRN) as well as cell-cell communication topologies with explicit causal directionality. The resulting synthetic data exhibits non-classical dependencies: standard correlation-based analyses (Pearson and Spearman) fail to recover the programmed causal pathways and instead report spurious associations driven by high baseline gene-expression probabilities. Furthermore, applying cell-cell communication detection to the simulated data validates the true mechanistic links, revealing a robust, up to 75-fold relative increase in inferred communication probability only when quantum entanglement is active. These results demonstrate that the quantum kernel is essential for producing high-fidelity ground-truth datasets and highlight the need for advanced inference techniques to capture the complex, non-classical dependencies inherent in gene regulation.

## 1 Introduction

Single-cell RNA sequencing (scRNA-seq) has transformed modern biology by enabling gene expression profiling at single-cell resolutions [1, 2]. This capability allows researchers to explore the molecular signatures that define individual cell identities and functions, thereby revealing the complexity of cellular heterogeneity. Gene expression, however, is not a linear process–it arises from intricate, nonlinear interactions among genes that form dynamic gene regulatory networks (GRNs) essential for cellular functions [3].

Simulating single-cell data is instrumental in developing and benchmarking computational approaches for understanding cellular heterogeneity. Existing tools to simulate single-cell behavior exist. However, generating realistic single-cell simulations remains challenging, as it requires capturing not only the intricate gene-gene regulatory dynamics within cells but also the ligand-receptor signaling that coordinates behavior across cells [4–6]. Indeed, recent benchmark studies highlight that classical simulators struggle to accommodate complex designs and yield unreliable performance estimates, emphasizing the need for more expressive models [7]. To address this gap, quantum generative modeling (QGM) is necessary to achieve the high expressivity required for complex probability distributions, offering an advanced approach to synthetic data generation [8, 9]. Most available simulators prioritize intracellular regulation, while models of cell-cell communication remain immature. For example, SERGIO and scMultiSim, are representative recent frameworks that have advanced the field by generating synthetic single-cell datasets that reflect gene regulatory dynamics [5, 6]. Meanwhile, attempts to model cell-cell communication remain rudimentary, typically approximating ligand–receptor signaling through static mappings with fixed communication probabilities [10, 11]. In a typical setup, to make two cells “interact”, the simulator goes through each sender cell and finds designated receiver cells within a neighborhood [12, 13]. For every pair of sender-receiver cells, it checks a list of matching ligand-receptor genes. Then, for each match, it slightly increases the receptor cell’s gene expression based on how strongly the sender cell expresses the ligand through linear correlation. This exhaustive, pairwise procedure must be repeated for every cell and gene pair, is slow, cumbersome, and provides no mechanistic intercellular feedback.

There is currently no platform capable of simulating both intracellular and intercellular dynamics in an integrated manner. We previously developed the quantum single-cell GRN framework (qscGRN), using a parameterized quantum circuit to infer GRNs from single-cell data [14]. By leveraging qubit entanglement to represent genegene dependencies and optimizing a Kullback-Leiber divergence-based loss function within a hybrid quantum-classical loop, qscGRN demonstrated the potential of quantum computing to uncover complex gene regulatory relationship beyond the reach of conventional statistical models [14]. Building upon this foundation, the present work introduces a hybrid quantum-classical simulator that extends quantum principles–specifically *superposition* and *entanglement*–to model single-cell gene expression and intercellular communication. In our design, qubits serve as analogues for genes or molecular features. Custom rotation gates initialize each qubit to represent basal gene expression levels, while CNOT gates introduce entangled relationships between qubits, thereby encoding the nonlinear topology of GRNs. This enables the generation of diverse and biologically realistic gene expression patterns unattainable by classical simulators. Furthermore, by entangling ligand and receptor gene qubits, our framework directly simulates cell–cell communication, capturing molecular crosstalk between distinct cell types. The key contribution of this study is a quantum-based simulation framework that models both intra-cellular regulatory mechanisms and inter-cellular signaling. By explicitly representing ligand-receptor interactions across different cell types, the proposed method provides a more direct and mechanistic view of cell-cell communication than classical neighborhood-based approaches.

## 2 Methods

**Fig. 1:**
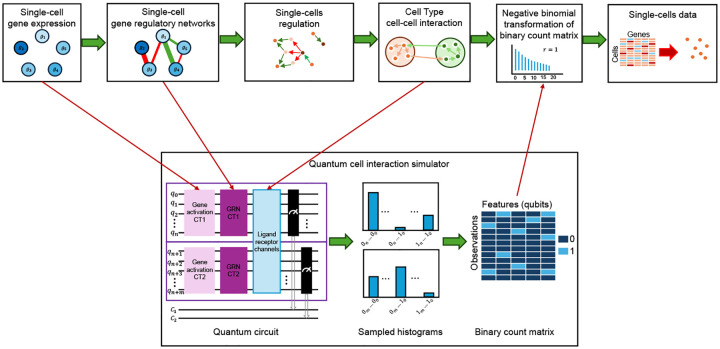
Quantum-simulated single-cell data framework. Our framework utilizes a quantum kernel, divided into three key sections, to generate realistic single-cell data. First, independent feature (gene) activation is simulated for two distinct cell types (CT1 and CT2). Second, a gene regulatory network (GRN) is established using controlled-NOT (CX) gates between source and target qubits, modeling gene-gene interactions. Third, inter-cellular (inter-state) communication channels are introduced and enhanced by CX gates, representing interactions between cell types. The simulation generates sampled binary histograms for CT1 and CT2, encoding complex inter- and intra-cellular communication within the feature (qubit) states. These histograms are then converted into a binary matrix, where each entry represents the simultaneous activation of features (genes) within individual simulated cells. The final step involves transforming this binary matrix into gene expression profile using a negative binomial augmentation, thus creating a more biologically realistic synthetic single-cell data.

We introduce qSimCells, as illustrated in Fig. 1, a quantum computing-based generative framework for single-cell data simulation.

The core of framework is a quantum kernel, designed to capture complex, non-classical dependencies through quantum entanglement. This kernel enables the simulation of both intra-cellular interactions within a quantum-entangled GRN and inter-cellular communication via ligand–receptor (LR) pair entanglement across distinct statevectors [14].

### 2.1 Quantum kernel of qSimCells

Quantum computing offers a very interesting perspective for modeling relationships between genes as well as between cells of different types [14]. Our proposition utilizes a Parameterized Quantum Circuit (PQC) where the initial cell state $\left|\psi_{0}\right\rangle$ is prepared using user-defined gene activation angles $\theta_{i}$.

The state initialization involves applying Y-axis rotation $\left(R_{y}\right)$ to each of the n qubits, which starts in the ground state |0⟩:

(1) $$\left|\psi_{0}\right\rangle ={⊗}_{i=0}^{n-1}R_{y}\left(\theta_{i}\right)\left|0_{i}\right\rangle .$$

Next, to integrate GRN interactions, we couple the expression of one gene to another using a Controlled-NOT (CX) gate. This process provides a unique entanglement inaccessible to classical computing, effectively pairing the target gene’s expression to reinforced activation or deactivation by the control gene (qubit) [14].

The resulting entangled state $\left|\psi_{1}\right\rangle$ is obtained by applying a sequence of MCX gates, where the sequence is crucial due to the non-commuting nature of the gates, potentially mimicking cascade activations:

(2) $$\left|\psi_{1}\right\rangle ={\left(\prod_{k=0}^{M-1}CX_{c_{k},t_{k}}\right)}_{\text{time-ordered}}\left|\psi_{0}\right\rangle .$$

The time ordered product means that the gates are applied sequentially, with $CX_{c_{1},t_{1}}$ applied first and $CX_{c_{M-1},t_{M-1}}$ applied last. The sequence of CX gates is explicitly defined by the list of control-target pairs $L={\left\{\left(c_{k},t_{k}\right)\right\}}_{k=0}^{M-1}$, which represents the GRN topology [14].

A key advantage of quantum computing lies in its ability to combine states via the tensor product, which drastically expands the Hilbert space and enables the representation of complex interactions [15]. More importantly, to simulate two distinct cell types, we can prepare two independent cell states, $\left|\psi_{1}\right\rangle$ (on n qubits) and $\left|\psi_{1}^{\prime }\right\rangle$ (on $n^{\prime }$ qubits), which are initially defined on disjoint sets of qubits. The combined state is formed by their tensor product:

(3) $$\left|\psi_{2}\right\rangle =\left|\psi_{1}\right\rangle ⊗\left|\psi_{1}^{\prime }\right\rangle .$$

The resulting composite state $\left|\psi_{2}\right\rangle$ lives in a Hilbert space of dimensions $2^{n+n^{\prime }}$ and serves as the foundation for modeling inter-state interactions (e.g., between different cell types) by applying subsequent entangling gates across the n and $n^{\prime }$ qubit registers, similar to the coupling defined in Eq. 2 [15]. Under the assumption that there is no interaction between two cell types (a baseline model), we keep the measurements for the two different cell state registers separated, allowing for independent analysis.

### 2.2 Final entanglement and quantum simulation

To complete the model, we introduce cell-cell interactions (between two cell types) by applying a final set of entangling gates. Those gates are put across the combined $\left|\psi_{2}\right\rangle$ state to establish inter-state interactions (e.g., cell-to-cell communication between the n and $n^{\prime }$ gene registers). This is achieved by applying a fixed sequence of KCX gates. The resulting final state, $\left|\psi_{\text{final}}\right\rangle$, is defined as:

(4) $$\left|\psi_{\text{final}}\right\rangle ={\left(\prod_{k=0}^{K-1}{\text{CX}}_{c_{k}^{\prime },t_{k}^{\prime }}\right)}_{\text{time-ordered}}\left|\psi_{2}\right\rangle .$$

The indices $c_{k}^{\prime }$ and $t_{k}^{\prime }$ here represent global qubit indices that span both the n and $n^{\prime }$ registers. The $\left|\psi_{\text{final}}\right\rangle$ state is obtained and executed using the Qiskit quantum computing framework developed by IBM [16]. Our methodology supports two implementations: for model prototyping, the circuit is run on the local quantum computer simulator, the AerSimulator; for realistic results incorporating quantum hardware noise, the circuit is executed on an IBM Quantum computer [16]. In both cases, the probability distribution of the final state is sampled by executing a fixed number of measurement shots using the SamplerV2 primitive. The number of shots, $N_{\text{shots}}$ is set equal to the total number of simulated single-cell observations m. Furthermore, the raw output bit strings from the simulation are reversed (e.g., $b=b_{n-1}\ldots b_{0}$) to align with the logical gene indices i=0⋯n-1, compensating for the little-endian ordering convention of the quantum simulator.

### 2.3 scRNA-seq count matrix generation

The simulation process begins by measuring the final quantum state $\left|\psi_{\text{final}}\right\rangle$ multiple times to obtain a histogram of the measurement outcomes. These outcomes are recorded into two distinct classical registers, allowing the probability distribution (marginalized for each cell state) to be assessed separately. This distribution is then used to assess the co-occurrence of gene activation (features) across m observation (simulated single-cells).

A binary count matrix $X^{\prime }$ is first constructed from this measurement histogram. Each measured bit string, $b=b_{0}b_{1}\ldots b_{n-1}$, corresponds to a single-cell observation where the value $b_{i}\in \{0,1\}$ indicates the deactivation or activation (expression) of gene i, respectively. If a bit string b has a measured count of C(b), it contributes C(b) rows to the n×m matrix $X^{\prime }$, where n is the number of genes. The binarization process can be formally represented by defining the matrix element $X_{ij}^{\prime }$ for gene i in cell j:

(5) $$X_{ij}^{\prime }=\left\{\begin{array}{ll} 1 & \text{if}\;\text{gene}\;i\;\text{is}\;‘\text{ON}’\;\text{in}\;\text{bit}\;\text{string}\;b_{j}\\ 0 & \text{if}\;\text{gene}\;i\;\text{is}\;‘\text{OFF}’\;\text{in}\;\text{bit}\;\text{string}\;b_{j} \end{array}\right.$$

The generated binary count matrix $X^{\prime }$ is then transformed to incorporate the continuous and noisy characteristics of gene expression counts observed in real single-cell data. This is achieved by multiplying the observed ‘ON’ states ($X_{ij}^{\prime }=1$) by a value sampled from the Negative Binomial distribution, a function commonly attributed to the overdispersed count data characteristic of scRNA-seq [17]. The final gene count matrix X is calculated as:

(6) $$X_{ij}=NB\left(r_{i},p_{i}\right)X_{ij}^{\prime }.$$

Here, $NB\left(r_{i},p_{i}\right)$ represents a random variate (or single random sample) drawn from the Negative Binomial (NB) distribution parameterized by gene-specific parameters, where $r_{i}$ (often related to dispersion) is the number of successful trials, and $p_{i}$ is the probability of success. $p_{i}$ is defined as $p_{i}=r_{i}/\left(\mu_{i}+r_{i}\right)$, where μ is the i-th gene mean. This transformation ensures that the final count $X_{ij}$ remains 0 if the gene was not activated in the quantum measurement ($X_{ij}^{\prime }=0$), but if $X_{ij}^{\prime }=1$, the expression level follows the stochastic, overdispersed behavior of a real gene. The parameters $r_{i}$ and $p_{i}$ are typically designed to mimic real scRNA-seq data to match the marginal statistics of the genes being modeled [7, 18].

### 2.4 Inferring gene regulatory networks with simulated data

To benchmark the complexity and non-classical nature of synthetic data, we applied classical GRN inference methods to the qSimCells simulated data. Prior to inference, the raw scRNA-seq count matrix X was preprocessed following standard single-cell analysis practices [19, 20]:

1. **Normalization:** Total counts were normalized to 10, 000 per cell to correct for sequencing depth differences.
2. **Transformation:** The data was ${\text{log}}_{1p}$-transformed to stabilize the variance and mitigate the influence of large count magnitudes [21].
3. **Scaling:** The data was standardized (Z-score scaled) per gene to ensure all features contributed equally to the correlation metrics.

Using this preprocessed matrix, gene-gene correlation matrices were computed across the cells using both the Pearson (linear) and Spearman (monotonic non-linear) correlation coefficients [22]. An adjacency matrix was then constructed by setting a strict threshold, retaining only edges where the absolute correlation value was greater than 0.5 (|Corr|>0.5). The resulting network topology was visualized using the NetworkX package, allowing for a direct comparison between the programmed quantum entanglement and the inferred classical dependencies [23].

## 3 Results

Our initial simulation demonstrates the capability of the proposed quantum kernel to model both intra-cellular regulations within (GRNs) and inter-cellular communication between distinct cell states. In this proof-of-concept study, we simulated a system consisting of five genes (n=5) for Cell Type 1 (CT1) and five genes ($n^{\prime }=5$) for Cell Type 2 (CT2) allowing us to evaluate the framework’s ability to capture gene-gene dependencies and LR-mediated cross-talk between cells.

### 3.1 Parameter initialization and qubit mapping

The initial self-activation level for each gene is set by its corresponding rotation angle $\theta_{i}$, as defined in Eq. 1. Since the $R_{y}$ gate maps 0 to the ‘OFF’ state and π to the ‘ON’ state (a full activation), the coefficients $p_{i}=\theta_{i}/\pi$ represent the proportional initial activation of each gene. These values are listed in Table 1.

To establish an unambiguous indexing system, the system’s $n+n^{\prime }=10$ qubits are mapped sequentially, following the augmentation in Eq. 3. This creates a global gene index $g_{i}$ (where i=0 to 9) that is identical to the qubit index $q_{i}$. Specifically:

- Genes $g_{0}$ to $g_{4}$ correspond to CT1.
- Genes $g_{5}$ to $g_{9}$ correspond to CT2.

We interchangeably utilize the global index notation $q_{i}↔g_{i}$ throughout the remainder of this work.

### 3.2 Entanglement and cascade activation

The entanglement topology governing both intra- and inter-state gene regulatory interactions was defined by the control-target list L. In this framework, intra-state interactions represent gene-gene regulation within a single cell type, while inter-state interactions represent ligand-receptor communication between two distinct cell types.

#### 3.2.1 Case 1: Inter-state cascade

In the first case, we designed a specific inter-state cascade utilizing the entanglement topology $L_{1}=\{(3,5),(5,7),(7,0)\}$. This configuration, applied in the $\left|\psi_{\text{final}}\right\rangle$ stage (Eq. 4), models a multi-step regulatory path spanning both cell types:

- **Step 1** (**CT1** →**CT2**): Gene $g_{3}^{\text{CT}1}$ (qubit $q_{3}$) activates gene $g_{0}^{\text{CT}2}$ (qubit $q_{5}$).
- **Step 2 (CT2 → CT2):** Gene $g_{0}^{\text{CT}2}$ (qubit $q_{5}$) activates gene $g_{2}^{\text{CT}2}$ (qubit $q_{7}$).
- **Step 3 (CT2 → CT1):** Gene $g_{2}^{\text{CT2}}$ (qubit $q_{7}$) activates gene $g_{0}^{\text{CT1}}$ (qubit $q_{0}$).

**Fig. 2:**
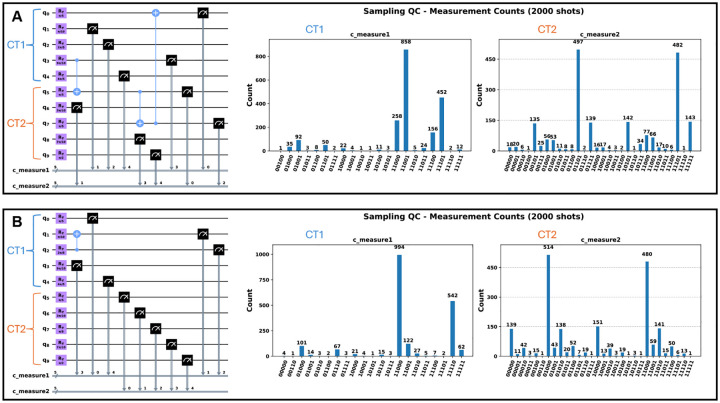
Quantum circuit sampling. A) shows inter-state interactions modeled by $L_{1}=\{(3,5),(5,7),(7,0)\}$ entanglement interactions on the quantum circuit, and the corresponding measurements per $\left|\psi_{0}\right\rangle$ state. B) shows non-communicating intra-state modeled by $L_{2}=\{(2,1)\}$ entanglement on the quantum circuit, and the corresponding measurements per $\left|\psi_{0}\right\rangle$ state.

The resulting entangled state $\left|\psi_{\text{final}}\right\rangle$ reflects this cascade. The entangling topology and the resulting measurement histogram are shown in Fig. 2A.

#### 3.2.2 Case 2: Non-interacting control

To highlight the effect of the inter-state communication, we performed a control experiment. We used the same activation angles (Table 1) but replaced the complex cascade with a simple, non-communicating intra-state entangler $L_{2}=\{(2,1)\}$. This design models an isolated regulation within CT1 (affecting $g_{2}^{\text{CT1}}$ and $g_{1}^{\text{CT1}}$) and enforces no communication between CT1 and CT2.

Fig. 2B shows the resulting measurement histogram for this non-cascading topology. A visual comparison between the histograms in Fig. 2A and 2B demonstrates how the programmed entangling topology significantly alters the final co-expression patterns.

### 3.3 Negative binomial augmentation for synthetic scRNA-seq data

Following the quantum simulation and measurement, the resulting $X^{\prime }$ matrix (the binary count matrix) is utilized to generate the final scRNA-seq count data, as described in Section 3.3 [17]. This step involves transforming the binary gene activation states into continuous count data by sampling from the NB distribution to accurately model the biological noise and overdispersion characteristic of single-cell sequencing [17].

For demonstration and simplified analysis, the gene-specific mean ($\mu_{i}$) and dispersion ($r_{i}$) parameters were uniformly set to $\mu_{i}=5$ and $r_{i}=1$ across all model genes in both cell types (CT1 and CT2). This modular approach ensures that the initial regulatory pattern is governed by the rotation angle $\theta_{i}$ and the GRN entanglement topology, while the final expression level (magnitude) and stochasticity are independently controlled by the customizable NB parameters $\mu_{i}$ and $r_{i}$.

To stabilize the cell type representations and provide a robust baseline for expression, we augmented the gene set by including 50 Housekeeping Genes (HKGs) [24]. These were assigned high, stable expression parameters: $\mu_{\text{HKG}}=80$ and $r_{\text{HKG}}=6$.

### 3.4 Effect of including inter-state interaction on simulated cell populations

**Fig. 3:**
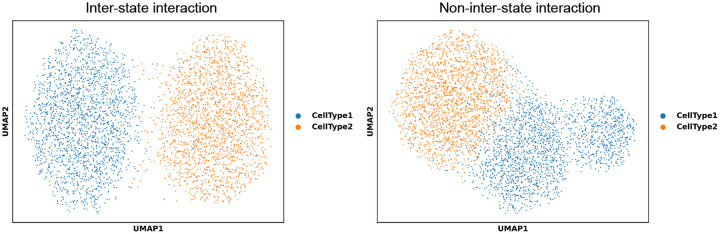
UMAP visualization of simulated scRNA-seq data. The UMAP plot displays the synthetic single-cell data generated under two different entanglement scenarios (Case 1: Inter-state cascade; Case 2: Non-interacting control), showing the resulting lineage separation between CT1 and CT2 populations.

The synthetic scRNA-seq data, visualized in the UMAP plot in Fig. 3, reveals a clear distinction between cell populations simulated with the two different entanglement scenarios: with and without inter-state interaction.

- **Inter-state cascade (case 1):** The data generated with the inter-state regulatory cascade shows a distinct separation of the CT1 and CT2 lineages in Fig. 3. This pronounced separation is due to two factors: the enforced cross-type expression enhancement, and the structural exclusion of gene expression, where CT2 genes ($q_{5}-q_{9}$) have zero expression in CT1 cells and, conversely, CT1 genes ($q_{0}-q_{4}$) have zero expression in CT2 cells [25]. This structural sparsity, combined with entanglement, drives the populations into structurally different high-dimensional states.
- **Non-interacting control (case 2):** Conversely, the non-interacting control experiment (where entanglement was restricted to only $L_{2}=\{(2,1)\}$) exhibits a more mixed or less pronounced separation. In this case, the expression pattern is primarily dominated by the uniformly highly expressed HKGs and the non-interacted model genes. The inter-state cascade is essential for providing the unique, non-linear expression patterns that maximize the separation of the cell states [25].

The observed lineage separation confirms that quantum entanglement, specifically when programmed to facilitate cross-state regulation, is the dominant factor shaping the distinct expression profiles of CT1 and CT2.

### 3.5 Synthetic scRNA-seq data analysis and classical predictions

The synthetic scRNA-seq data produced from quantum computing kernel has complex relationships that would be hardly embedded from classical regime, but we can still see if from classical regime, we could get the grasp of what was embedded. To this purpose, we propose two analyses, one being the GRN through correlation methods, e.g., Pearson and Spearman. While the second is to apply CellChat to see if we can get the cell communication difference between the two previous cases.

#### 3.5.1 Inferred gene regulatory networks from synthetic data

**Fig. 4:**
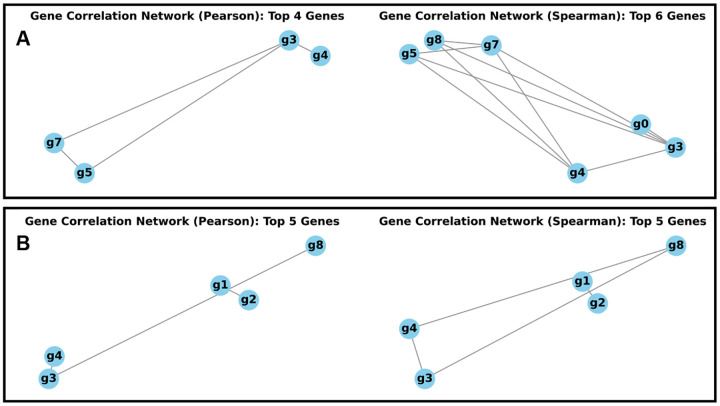
Gene regulatory networks through classical prediction. A) shows GRN inference through Pearson and Spearman correlation computed adjacency matrix above 0.5 threshold for interstate interaction simulation. B) shows GRN inference through Pearson and Spearman correlation computed adjacency matrix above 0.5 threshold for non-inter-state simulation.

The classical network inference results, presented in Fig. 4, compare the predicted GRNs against the programmed quantum entanglement topologies. The networks were constructed using a strong absolute correlation threshold of ∣Corr∣>0.5 on the fully preprocessed (normalized, ${\text{log}}_{1p}$-transformed, and scaled) synthetic data.

The analysis reveals that both Pearson and Spearman correlation methods, despite filtering out the background “noise” (HKGs), do not recover the programmed quantum entanglement topology but instead report emergent correlation structures among the model genes [26]. The computed adjacency matrices are highly fragmented and structurally distinct from the CX paths:

##### Case 1: Inter-state cascade

The programmed quantum cascade was $q_{3}\to q_{5}\to q_{7}\to q_{0}$. The classical networks yield connections only among the model genes, successfully excluding the highmagnitude HKGs. However, they report relationships that are emergent effects of the quantum state, not the direct CX links:

- The Pearson network (left, Fig. 4A) identifies strong correlations among $g_{3},g_{4},g_{5}$, and $g_{7}$. The structure forms a single, fragmented component. This high correlation is interpreted as a coincidental statistical artifact driven by the differential initial within CT1 and CT2, specifically:
  1. The correlation is primarily influenced by the single dominant initial activation angle ($\theta_{4}=0.9\pi$) of gene $g_{4}$. This high base probability of being ‘ON’ creates a statistical tendency to co-occur strongly with its neighbors $\left(g_{3},g_{5},g_{7}\right)$, regardless of the true quantum causal path.
  2. The network is structurally incorrect (e.g., omitting the final target gene, $g_{0}$), demonstrating that Pearson’s method prioritizes emergent statistical co-occurrence resulting from high base probability over the subtle causal changes introduced by the CX entangling gates.
- The Spearman network (right, Fig. 4A) finds a higher density of non-linear monotonic dependencies [26], resulting in a highly interlinked graph involving $g_{0},g_{3},g_{4},g_{5},g_{7},g_{8}$. This complex structure does not resemble the targeted regulatory path but confirms that the entanglement creates widespread, complex non-linear statistical co-dependencies among the model genes.

##### Case 2: Non-interacting control

The simple programmed link was $q_{2}\to q_{1}$. The networks are sparse and report almost no meaningful inter-gene connections. The initial conditions for the programmed genes are $\theta_{2}=0.4\pi$ and $\theta_{1}=0.1\pi$, while the strongest self-activations are on $g_{3}\left(\theta_{3}=0.9\pi \right),\;g_{4}\left(\theta_{4}=0.8\pi \right)$ and $g_{8}\left(\theta_{8}=0.7\pi \right)$ :

- The Pearson network (left, Fig. 4B) is minimal, identifying two isolated pairs: $g_{1}-g_{2}$ and $g_{3}-g_{4}$, with $g_{8}$ also present as an isolated node. Crucially, the programmed link $q_{2}\to q_{1}$ (the $g_{2}\to g_{1}$ connection) is recovered as a strong correlation between $g_{1}$ and $g_{2}$. However, the strong $g_{3}-g_{4}$ link represents a spurious correlation driven by the high base activation of those genes.
- The Spearman network (right, Fig. 4B) finds a similarly sparse and highly fragmented structure [26]. While it recovers the $g_{1}-g_{2}$ connection, the remaining links are likely coincidental correlations driven by the differential self-activation angles $\left(\theta_{i}\right)$ within cell types rather than genuine entanglement effects. For instance, the genes $g_{3}$ and $g_{4}$ form an isolated pair, and $g_{8}$ remains an isolated node, confirming that the majority of connections are artifacts stemming from the initial high-probability states.

##### Interpretation: Quantum causality vs. classical correlation

The analysis confirms a critical distinction between the programmed quantum causality and the emergent classical correlations. The consistent finding is that the classical methods successfully filtered out the background noise (HKGs) but only captured partially correct interactions and failed to reconstruct the complete programmed CX paths.

The quantum kernel establishes causal dependencies ($CX_{i,j}$) which, through superposition and measurement, result in a complex joint probability distribution. The final classical correlations are not the direct causal links, but rather emergent statistical relationships created by the entanglement on the sampled state.

- **High-probability bias:** The Pearson method, in the inter-state cascade (case 1), was heavily influenced by the single dominant initial activation angle ($\theta_{4}=0.9\pi$) of $g_{4}$. This led to coincidental statistical artifacts–structurally incorrect correlations $\left(g_{3}-g_{4}\right)$–that overshadowed the true quantum links, demonstrating the method’s vulnerability to high base probability over subtle quantum signals.
- **Incomplete recovery:** While the simple programmed link $\left(q_{2}\to q_{1}\right)$ was successfully recovered in the non-interacting control (case 2), the presence of other connections (e.g., $g_{3}-g_{4}$) are artifacts of the non-interacting genes’ high base activation angles $\left(\theta_{i}\right)$. This confirms that the methods are easily biased by varying initial probabilities, resulting in misleading correlation structures.

This demonstrates that the quantum-generated data does not represent a simple sum of linear or monotonic pairwise relationships, but rather exhibits non-classical dependencies that fundamentally challenge standard correlation-based classical analysis tools, even when rigorous preprocessing is applied [27].

#### 3.5.2 Detecting cell-cell interactions with synthetic data

We applied CellChat, a widely used classical method of cell-cell interaction detection, to infer cell-cell communication patterns from synthetic scRNA-seq data. CellChat predicts intercellular signaling by modeling interaction probabilities of LR pairs [13]. Our objective was to evaluate whether CellChat could accurately recover the true mechanistic interactions embedded within the simulated data while distinguishing them from spurious correlations driven by highly expressed but non-interacting genes.

In our experimental setup, the custom LR database contained two categories of interactions (1) True inter-state interactions ($g_{3}\to g_{5}$ and $g_{7}\to g_{0}$), which represent the mechanistic LR pairs responsible for genuine intercellular signaling; and (2) False control pairs $\left(g_{8}\to g_{4}\right.$ and $\left.g_{9}\to g_{4}\right)$, which were included to evaluate CellChat’s ability to discriminate between authentic and coincidental association. Details of these four simulated LR interactions are provided in Table 2.

The results presented in Table 3, validate that the proposed gene activation patterns (from Table 1) and the corresponding entanglement topologies ($L_{1}$ and $L_{2}$) provide strong mechanistic consistency with the designed regulatory framework. As anticipated, the true LR pairs exhibited substantial increase in inferred communication probabilities when the inter-state interactions were activated. For instance, the LR pair $g_{3}\to g_{5}$ increased from 0.0148 (non-interacting) to 0.1135 (inter-state interaction), representing more than a 7-fold enhancement. Similarly, $g_{7}\to g_{0}$ exhibited an even greater amplification-approximately a 75-fold increase-indicating a pronounced activation of cross-cell signaling. These findings are fully consistent with our design principle, in which entanglement topologies were orchestrated by the master gene regulator, $g_{3}$, thereby validating the role of quantum entanglement in modeling mechanistic intercellular communication.

Conversely, the false LR pairs, which were included in the custom database to represent coincidentally co-expressed but non-mechanistic genes, showed minimal changes in inferred communication probability. This outcome aligns with expectations, as no direct entanglement (mechanistic link) was set for these pairs. For instance, the communication probability for $g_{9}\to g_{4}$ showed only a marginal change, shifting from 0.0726 (non-interacting) to 0.0638 (interacting), corresponding to a ratio of 0.88 (approximately unchanged). This result confirms that the inference method effectively distinguished true mechanistic links from spurious co-activation driven by expression magnitude. Crucially, while the method still assigned high confidence probability (with p-value=0) even in the absence of mechanistic coupling, such absolute measures can be misleading. The relative change in communication probability between the interacting and no-interacting states, not the absolute probability or p-value, emerged as the more reliable indicator for reliably validating the synthetic mechanistic links [28].

## 4 Discussion

To conclude, our work introduces qSimCells, a novel hybrid quantum-classical simulator developed to address the fundamental challenge of generating realistic scRNA-seq data. A recent benchmark study has demonstrated that classical methods struggle with complex designs and yield unreliable performance estimates [7], justifying the need for a new approach. To this end, quantum generative models are uniquely positioned to handle the complex and highly correlated probability distributions required for realistic biological data, leveraging the exponentially large Hilbert space of quantum registers [8]. By leveraging quantum entanglement, qSimCells encodes complex, nonlinear topologies of GRNs and cell-cell communication. To the best of our knowledge, qSimCells is the only computational tool that simulates intracellular and intercellular dynamics in a natural and integrated manner. The central advantage of our quantum kernel lies in its ability to enforce causal dependencies through the time-ordered application of CX gates [15], thereby establishing explicit directionality and true cause-effect relationships. This enables the modeling of biologically meaningful cascades such as $g_{3}\to g_{5}\to g_{7}\to g_{0}$, which are impossible to capture using classical, correlation-based simulators [7].

Our results demonstrate that the synthetic datasets generated by qSimCells exhibit non-classical dependencies, making them an ideal ground truth for benchmarking advanced inference algorithms. When classical GRN inference methods were applied to the quantum-generated data, they failed to reconstruct the intended CX-based casual paths [29]. Instead, they produced spurious statistical associations—relationships arising from the global probability distribution rather than discrete, mechanistic links (Fig. 4). Classical models were particularly sensitive to baseline expression probabilities determined by initial gene activation angles $\left(\theta_{i}\right)$, often overemphasizing correlations such as $g_{3}-g_{4}$ that overshadowed the true, entanglement-induced dependencies [7]. These findings confirm that classical correlation-based approaches inherently lack directionality, prioritizing expression magnitude over casual order and therefore cannot recover the true underlying structure encoded by the quantum kernel.

Application of CellChat to the inter-state communication data provided strong validation of our quantum-derived ground truth while also revealing methodological limitations of cell-cell communication inference frameworks [13]. The tool successfully identified the true LR pairs ($g_{3}\to g_{5}$ and $g_{7}\to g_{0}$) with a 7- to 75- fold increase in communication probability under interacting conditions, consistent with the programmed entanglement. However, the false LR pairs, which lacked mechanistic links, were still assigned high-confidence probabilities (e.g., p-value=0). Such absolute confidence, in the absence of mechanistic coupling, can be misleading unless compared across experimental conditions. Therefore, our results highlight that the relative change in communication probability-between interacting and non-interacting state—serves as a more reliable indicator of true causal signaling than absolute probability or p-values.

In conclusion, qSimCells represents a quantum-enhanced platform for generating high-fidelity synthetic scRNA-seq data. Through quantum entanglement, it establishes a controlled and interpretable ground truth characterized by directionality, nonlinearity and causal coherence–features that are largely inaccessible to classical models. This framework not only exposes the inherent weakness of traditional inference techniques but also offers a new paradigm for developing algorithms capable of capturing the non-classical dependencies intrinsic to gene regulation. Ultimately, our results confirm that the quantum kernel is essential for constructing benchmark datasets in which the causal architecture is unequivocally defined, paving the way for next-generation computational methods that move beyond linear correlations toward a truly mechanistic understanding of cellular systems.

Finally, our findings argue for a quantum-native, generative paradigm in machine learning [8, 9], particularly for single-cell modeling. Quantum circuits naturally encode joint probability distributions through superposition and entanglement, making them well-suited to synthesize transcriptomes that embed nonlinear, causal gene-gene and cell-cell dependencies. qSimCells exemplifies this advantage. In contrast, quantum machine learning that simply mirrors classical discriminative pipelines and losses—designed for classical hardware—fails to exploit quantum advantages and often incurs prohibitive readout and training overhead. Thus, generative quantum models should be the primary path forward, coupled with quantum-native objectives and inference strategies capable of capturing the non-classical dependencies inherent in gene regulation.

## Supplementary Material

All required materials are included within the manuscript and GitHub.

## Figures and Tables

**Table 1:** Initial gene activation parameters and qubit mapping

Global Index ($q_{i}/g_{i}$)	Activation $p_{i}=\theta_{i}/\pi$	Cell Type	Local Gene Index
$q_{0}\equiv g_{0}$	0.2	CT1	$g_{0}^{\text{CT}1}$
$q_{1}\equiv g_{1}$	0.1	CT1	$g_{1}^{\text{CT}1}$
$q_{2}\equiv g_{2}$	0.4	CT1	$g_{2}^{\text{CT}1}$
$q_{3}\equiv g_{3}$	0.9	CT1	$g_{3}^{\text{CT}1}$
$q_{4}\equiv g_{4}$	0.8	CT1	$g_{4}^{\text{CT}1}$
$q_{5}\equiv g_{5}$	0.2	CT2	$g_{0}^{\text{CT}2}$
$q_{6}\equiv g_{6}$	0.3	CT2	$g_{1}^{\text{CT}2}$
$q_{7}\equiv g_{7}$	0.2	CT2	$g_{2}^{\text{CT}2}$
$q_{8}\equiv g_{8}$	0.7	CT2	$g_{3}^{\text{CT}2}$
$q_{9}\equiv g_{9}$	0.5	CT2	$g_{4}^{\text{CT}2}$

**Table 2:** Simulated Ligand-Receptor interactions

Interaction Name	Pathway	Ligand	Receptor	Annotation	Evidence
g3_g5_simulated	Simulated1	g3	g5	Secreted Signaling	Simulated1
g7_g0_simulated	Simulated2	g7	g0	Secreted Signaling	Simulated2
g8_g4_simulated	Simulated3	g8	g4	Secreted Signaling	Simulated3
g9_g4_simulated	Simulated4	g9	g4	Secreted Signaling	Simulated4

**Table 3:** CellChat Ligand-Receptor Inference with Simulated Pairs

Source	Target	Ligand	Receptor	Prob.	p-val	Pathway	Dataset
CT1	CT2	g3	g5	0.0148	0	Simulated1	Non-interacting
CT2	CT1	g7	g0	0.0011	0	Simulated2	Non-interacting
CT2	CT1	g8	g4	0.1133	0	Simulated3	Non-interacting
CT2	CT1	g9	g4	0.0726	0	Simulated4	Non-interacting
CT1	CT2	g3	g5	0.1135	0	Simulated1	Inter-state interaction
CT2	CT1	g7	g0	0.0830	0	Simulated2	Inter-state interaction
CT2	CT1	g8	g4	0.1008	0	Simulated3	Inter-state interaction
CT2	CT1	g9	g4	0.0638	0	Simulated4	Inter-state interaction

## Data Availability

Our algorithm is publicly available on GitHub https://github.com/cailab-tamu/qSimCells.
